# Chagas Disease among the Latin American Adult Population Attending in a Primary Care Center in Barcelona, Spain

**DOI:** 10.1371/journal.pntd.0001135

**Published:** 2011-04-26

**Authors:** Carme Roca, María Jesús Pinazo, Paolo López-Chejade, Joan Bayó, Elizabeth Posada, Jordi López-Solana, Montserrat Gállego, Montserrat Portús, Joaquim Gascón, and the Chagas-Clot Research Group

**Affiliations:** 1 El Clot Primary Care Center, Institut Català de la Salut, Universitat de Barcelona, Barcelona, Spain; 2 Barcelona Centre for International Health Research (CRESIB), Hospital Clinic-Universitat de Barcelona, Barcelona, Spain; 3 Parasitology Laboratory, Facultat de Farmàcia, Universitat de Barcelona, Barcelona, Spain; National Institutes of Health, United States of America

## Abstract

**Background/Aims:**

The epidemiology of Chagas disease, until recently confined to areas of continental Latin America, has undergone considerable changes in recent decades due to migration to other parts of the world, including Spain. We studied the prevalence of Chagas disease in Latin American patients treated at a health center in Barcelona and evaluated its clinical phase. We make some recommendations for screening for the disease.

**Methodology/Principal Findings:**

We performed an observational, cross-sectional prevalence study by means of an immunochromatographic test screening of all continental Latin American patients over the age of 14 years visiting the health centre from October 2007 to October 2009. The diagnosis was confirmed by serological methods: conventional in-house ELISA (cELISA), a commercial kit (rELISA) and ELISA using *T cruzi* lysate (Ortho-Clinical Diagnostics) (oELISA). Of 766 patients studied, 22 were diagnosed with *T. cruzi* infection, showing a prevalence of 2.87% (95% CI, 1.6–4.12%). Of the infected patients, 45.45% men and 54.55% women, 21 were from Bolivia, showing a prevalence in the Bolivian subgroup (n = 127) of 16.53% (95% CI, 9.6–23.39%).

All the infected patients were in a chronic phase of Chagas disease: 81% with the indeterminate form, 9.5% with the cardiac form and 9.5% with the cardiodigestive form. All patients infected with *T. cruzi* had heard of Chagas disease in their country of origin, 82% knew someone affected, and 77% had a significant history of living in adobe houses in rural areas.

**Conclusions:**

We found a high prevalence of *T. cruzi* infection in immigrants from Bolivia. Detection of *T. cruzi*–infected persons by screening programs in non-endemic countries would control non-vectorial transmission and would benefit the persons affected, public health and national health systems.

## Author Summary

Chagas disease is a parasitic infection caused by the protozoan *Trypanosoma cruzi*, and is becoming an emerging health problem in non-endemic areas because of growing population movements. The clinical manifestations of chronic *T. cruzi* infection include the latent form (the indeterminate chronic form), the cardiac form, the digestive or cardiodigestive form, and sudden death. Therefore, many diagnoses of Chagas disease are based on epidemiological suspicion rather than on clinical signs and symptoms. This study showed that the prevalence of Chagas disease in Latin American patients attending at a health center in Barcelona is 2,87% and the highest prevalence was found among Bolivian patients (16,53%). All the infected patients were in a chronic phase of Chagas disease. Detection of *T. cruzi*–infected persons by screening programs in non-endemic countries would control non-vectorial transmission and would benefit the persons affected, public health and national health systems. The data obtained in this study and the experiences described elsewhere suggest that it is advisable to perform Chagas disease screening in non-endemic countries on all patients from continental Latin America who: (1)have a suggestive epidemiologic history, (2)are pregnant, (3)are immunosuppressed, (4)have symptoms suggestive of Chagas disease, or (5)request screening.

## Introduction

*Trypanosoma cruzi* (*T. cruzi*) is a flagellate protozoan that causes Chagas disease (CD). It is traditionally linked to rural areas of continental Latin America, where it is transmitted by a variety of bug vectors. In recent decades, the epidemiological pattern of this disease has undergone considerable changes [1]. In the endemic countries of Latin America, the regional Chagas programs are working to interrupt vector-borne and transfusional transmission, to control congenital Chagas disease and to support initiatives aimed at improving diagnosis, management and surveillance of the disease [2]. In non-endemic countries that receive immigrants from Latin America or send tourists to endemic areas, CD is an emerging disease and has become a public health problem because it can be transmitted by non-vectorial mechanisms [3], [4].

Spain is a major European host country for people from Latin America. According to the Spanish National Institute of Statistics, in 2009 more than 1.8 million immigrants from Latin America were registered, accounting for 3.85% of the total population [5]. In recent years several studies of CD in non-endemic countries [6]–[9] have focused in particular on non-vectorial transmission mechanisms such as pregnancy and childbirth [10], [11], blood transfusion [12], [13] and organ transplantation [14], [15]. However, when reviewing the literature we found little information on imported CD in non-endemic countries at the primary care level [16], which is ideal for screening the general population [17].

The clinical manifestations of chronic *T. cruzi* infection include the latent form (the indeterminate chronic form), which occurs in 60% of cases [18], the cardiac form [19], the digestive or cardiodigestive form, and sudden death [20]. Therefore, many diagnoses of CD are based on epidemiological suspicion rather than clinical signs and symptoms.

The objectives of the present study were (1) to assess the prevalence of *Trypanosoma cruzi* infection in the adult Latin American population treated at a health center in Barcelona, Spain; (2) to analyze the clinical phase of the disease; and (3) to determine whether screening for imported CD in primary care should be recommended.

## Methods

### Study design and scope

We performed an observational, cross-sectional prevalence study at the health center of the Clot district, Barcelona. This center serves a population of 25442 people, with a total foreign population of 13.5% and a Latin American population of 6.3% (according to the 2008 census of the Barcelona City Council) [21]. The staffs participating in the study were 14 general practitioners, 13 nurses, 1 gynecologist and 1 midwife.

### Ethical conduct

The study protocol was approved by the Ethical Committee of the Jordi Gol Institute for Research in Primary Care of the *Institut Català de la Salut* (Catalan Health Institute).

Written informed consent was requested from all participants. When participants were children, their parents/guardians provided informed consent.

### Patients

During the period October 2007 to October 2009, all patients from continental Latin America under 14 years of age who presented at the health center for any health reason were invited to participate in the study. After obtaining informed consent, we collected clinical and epidemiological data.

We ascertained the reasons why the patients visited their doctor/nurse by reviewing the electronic patient charts. On a patient's first visit to a primary care centre the Preventive Activities and Health Promotion Program (PAPPS) is initiated [22], and at the Clot health center this program included CD screening of all persons originating from continental Latin America.

### Serological techniques

Serological screening was performed with an immunochromatographic test (ICT) that uses recombinant antigens of *T. cruzi* (TcD, TcE, PEP-2 and SAPA) on whole blood collected by finger prick.

If the screening was positive, a venous blood sample was collected to confirm the diagnosis at the Parasitology Laboratory of the Faculty of Pharmacy, University of Barcelona. We used 2 enzyme-linked immunosorbent assay (ELISA) methods: a conventional, in-house ELISA (cELISA) with whole *T. cruzi* epimastigote antigens [23] and a commercial kit with the recombinant antigens TcD, TcE, PEP-2 and TcLo1.2 (rELISA).

In accordance with international criteria established by the World Health Organization, sera that were reactive in two serological methods were considered positive [24]. Positive results were confirmed by a third ELISA using *T cruzi* lysate (Ortho-Clinical Diagnostics) (oELISA). In a subsample of 101 patients we performed the ELISA serologies (cELISA and rELISA) regardless of the result of the ICT, in order to test the usefulness of this test in screening for CD in primary care [25].

### Management of patients

All patients infected with *T. cruzi* were referred to the Tropical Medicine Unit of Hospital Clínic de Barcelona and clinically evaluated by a complete review of the epidemiologic history and consistent symptoms/signs, a general physical examination and an electrocardiogram. If the electrocardiogram was pathologic, it was assessed with an echocardiogram or 24-hour Holter according to the disease detected. If symptoms consistent with gastrointestinal involvement were detected [26], an esophageal, gastric and duodenal transit assessment or a barium enema was performed. Benznidazole (5 mg/kg/day for 60 days) was offered to all patients aged 18–50 years without advanced Chagas cardiomyopathy and no other contraindication for start benznidazole (pregnancy, severe renal or hepatic insufficiency [27]. None of them refused to start it.

### Statistical calculations

The sample size was calculated for an alpha level of 0.05 and a precision of ±0.05% in a bilateral comparison, assuming maximum uncertainty (50% prevalence); for a population of 1516 subjects [28] a random sample of 758 was necessary.

The programs SPSS version 17.0 and Epidat version 3.1 were used for the statistical analysis. The χ2 test was used to compare hypotheses of independence between two categorical variables and the Student *t* test for continuous variables. The confidence interval for all hypothesis comparisons was 95% and the tests were 2-tailed.

## Results

A total of 766 persons from continental Latin America were included in the study. The epidemiological data are presented in Table 1 and the countries of origin in Table 2.

**Table 1:** Epidemiologic data.

Variables	Without CD n = 744	With CD n = 22	p, statistical significance
MenWomen	296 (39.8%)448 (60.2%)	10 (45.45%)12 (54.5%)	Not statistically significant (NS)
Age; years (SD)	36.43 (12.2)	39.86 (9.88)	NS
Journeys to country of origin in last 12 months	Yes 303 (40.7%)No 441 (59.3%)	Yes 6 (27.27%)No 16 (72.73%)	NS
Had lived in rural areas	Yes 248 (33.3%)No 496 (66.7%)	Yes 17 (77.22%)No 5 (22.73%)	p<0.001
Had lived in adobe houses	Yes 166 (22.3%)No 578 (77.7%)Don't know —	Yes 17 (77.3%)No 4 (18.2%)Don't know 1(4.5%)	p<0.0001
Had received transfusion in country of origin	Yes 53 (7.1%)No 691 (92.9%)	Yes 3 (13.64%)No 19 (86.36%)	NS
Had heard of CD in country of origin	Yes 317 (42.6%)No 427 (57.4%)	Yes 22 (100%)No 0 (0%)	p<0.0001
Knew someone with CD	Yes 89 (12%)No 655 (88%)	Yes 18 (81.81%)No 4 (18.19%)	p<0.0001

**Table 2:** Countries of origin.

Country	Total n = 766 (%)	Infected by *T. cruzi* n = 22 (%)
Peru	173 (22.9)	
Ecuador	171 (22.3)	
Bolivia	127 (16.6)	21 (95.45)
Colombia	102 (13.3)	
Argentina	63 (8.2)	
Venezuela	31 (4)	
Brazil	23 (3)	
Chile	22 (2.9)	
Paraguay	19 (2.5)	1 (4.55)
Uruguay	14 (1.8)	
Honduras	12 (1.6)	
El Salvador	5 (0.5)	
Mexico	2 (0.2)	
Guatemala	1 (0.1)	
Panama	1 (0.1)	

Of the 766 patients analyzed, 27 were reactive to the ICT and 20 of these were reactive in cELISA, rELISA and oELISA. Also, 2 patients of the 101 tested by ICT, cELISA, and rELISa regardless of the result of the first were reactive in cELISA and rELISA. Both were also positive in oELISA, so they were considered positive.

A total of 22 patients were diagnosed with CD, corresponding to a prevalence of 2.87% (95% CI, 1.6–4.12%) in the sample studied. Of these, 21 were from Bolivia; the prevalence of CD in the subgroup of Bolivian patients studied (n = 127) was 16.53% (95% CI, 9.6–23.39%). The remaining patient was from Paraguay.

All the patients infected by *T. cruzi* were in the chronic phase of CD. The clinical form and the reasons why they visited the health center are presented in Table 3. Four patients (18.2%) had been previously diagnosed in the country of origin, but none of them mentioned it in the primary care visit because they thought it was a health problem proper to their country that would be unknown to the Spanish health staff (this information was obtained when they were asked for informed consent to participate in the study). None of them were aware of their clinical phase and 2 patients had received incomplete treatment.

**Table 3:** Clinical phase of Chagas disease (CD) and reasons for visit.

	Clinical symptoms not suggestive of CD	Clinical symptoms suggestive of CD	PAPPS^a^		Total no. of patients with *T. cruzi* infection and complete studyn = 21^b^
			Diagnosed in country of origin	Not previously diagnosed	
Indeterminate form^18^	8	1^c^	3	5	17 (81%)
Cardiac form^19^	1	-	1	-	2 (9.5%)^e^
Digestive form^26^	-	-	-	-	0 (0%)
Cardiodigestive form	-	1^d^	-	1	2 (9.5%)^f^
	9 (42.8%)	2 (9.5%)	4 (19.1%)	6 (28.6%)	21 (100%)

a PAPPS: Preventive Activities and Health Promotion Program.

b Of the 22 patients diagnosed with CD, one failed to complete the tests.

c Dysphagia dating from >1 year earlier.

d Longstanding palpitations and dysphagia.

e One case with right bundle Branch block, and other with a T-wave inversion in inferior leads.

f One case: right bundle branch block associated with left anterior hemiblock and hypotonia of the lower esophageal sphincter. Second case: right bundle branch block with severe hypotonia of the lower esophageal sphincter and distal esophageal hypoperistalsis.

## Discussion

The prevalence of *T. cruzi* infection in the sample studied was 2.87% and in the subgroup of Bolivian patients it was 16.53%. In the medical literature we found few studies of similar characteristics to ours (involving screening of the adult Latin American population in primary care) and their results varied [29], [12] due to the heterogeneity of the populations analyzed and the distribution of CD in Latin America.

The laboratory confirmation of a clinical suspicion of CD is based on consistent results of at least 2 different immunological tests [24]. ICTs are attractive in primary care because they are easy to use in routine clinical practice and do not require sophisticated facilities or specialized staff. In the substudy that we performed in 101 patients [25], for the ICT used we found a sensitivity of 92.5% and a specificity of 96.8%. Other studies have evaluated the sensitivity and specificity of ICTs [30], [31] with similar results. The current sensitivity of ICTs must be increased so that they can be used as effective screening tests. Meanwhile, they should be combined with other methods that offer greater sensitivity [25], [31].

The highest prevalence was found among Bolivian patients, in agreement with other studies performed in Spain [9], [16] and other non-endemic countries [12], [32]. No cases were diagnosed among the Peruvian or Ecuadorian patients, who formed 45% of the sample, probably due to the heterogeneous distribution of CD in endemic countries and the lower seroprevalence of *T. cruzi* estimated in Peru (0.69%) and Ecuador (1.74%) [33].

An epidemiologic history of having lived in rural areas and/or adobe houses showed a significant relationship with *T. cruzi* infection, consistent with the dominant vector-borne transmission mechanism in the countries of origin. All patients with *T. cruzi* infection had heard of CD in their countries of origin and approximately 82% knew someone who was affected. These data should be taken into account for establishing CD screening criteria in immigrants from endemic zones, because mere knowledge of the disease may be considered as an indirect indicator of its presence in the region of origin.

In non-endemic countries CD screening programs have been aimed at particularly susceptible groups: in blood banks (France [34], the USA [35] and Spain [36]), and in pregnant Latin American women and their neonates (the Spanish autonomous communities of Catalonia [37] and Valencia [38]). In our study only 9.5% of the patients with *T. cruzi* infection had visited the health center due to clinical symptoms suggestive of CD. As it is a silent disease that has recently appeared in non-endemic countries, we stress the importance of establishing in these countries health screening programs based on compatible epidemiologic history among the general immigrant population from endemic areas. These programs should be multidisciplinary [3], supported by the best scientific evidence possible, and promoted by the health authorities.

In non-endemic countries, detecting persons infected by T. cruzi is important in order to control the transmission (vertical, by transfusion, or by organ transplant), reduce reactivations in immunodepressed persons, and delay the onset of the chronic cardiac form through antiparasite treatment [39], all of which have a great impact on the persons affected, on public health, and on health systems. Nevertheless, the best solution for CD is a combination of treatment and prevention in endemic countries [40], [41], where many programs and initiatives are underway [2], [42].

The data obtained in this study and the experiences described elsewhere [4], [12], [14], [16], [20], [27] suggest that it is advisable to perform CD screening in non-endemic countries on all patients from continental Latin America who: (1) have a suggestive epidemiologic history (having lived in a rural area, in adobe houses or having knowledge of CD in the country of origin), (2) are pregnant, (3) are immunosuppressed, (4) have symptoms suggestive of CD, or (5) request screening.
